# Non-Destructive Detection of Soybean Storage Quality Using Hyperspectral Imaging Technology

**DOI:** 10.3390/molecules30061357

**Published:** 2025-03-18

**Authors:** Yurong Zhang, Wenliang Wu, Xianqing Zhou, Jun-Hu Cheng

**Affiliations:** 1 School of Food and Strategic Reserves, Henan University of Technology, Zhengzhou 450001, China; yurongzh@163.com (Y.Z.); wuqingjianwww@163.com (W.W.); 2 Engineering Research Center of Grain Storage and Security of Ministry of Education, Zhengzhou 450001, China; 3 Henan Provincial Engineering Technology Research Center on Grain Post Harvest, Zhengzhou 450001, China; 4 School of Food Science and Engineering, South China University of Technology, Guangzhou 510641, China

**Keywords:** soybean, crude fatty acid values, storage quality, hyperspectral imaging, chemical information visualization

## Abstract

(1) Background: Soybean storage quality is crucial for subsequent processing and consumption, making it essential to explore an objective, rapid, and non-destructive technology for assessing its quality. (2) Methods: crude fatty acid value is an important indicator for evaluating the storage quality of soybeans. In this study, three types of soybeans were subjected to accelerated aging to analyze trends in crude fatty acid values. The study focused on acquiring raw spectral information using hyperspectral imaging technology, preprocessing by the derivative method (1ST, 2ND), multiplicative scatter correction (MSC), and standard normal variate (SNV). The feature variables were extracted by a variable iterative space shrinkage approach (VISSA), competitive adaptive reweighted sampling (CARS), and a successive projections algorithm (SPA). Partial least squares regression (PLSR), support vector machine (SVM), and extreme learning machine (ELM) models were developed to predict crude fatty acid values of soybeans. The optimal model was used to visualize the dynamic distribution of these values. (3) Results: the crude fatty acid values exhibited a positive correlation with storage time, functioning as a direct indicator of soybean quality. The 1ST-VISSA-SVM model was the optimal predictive model for crude fatty acid values, achieving a coefficient of determination (R^2^) of 0.9888 and a root mean square error (RMSE) of 0.1857 and enabling the visualization of related chemical information. (4) Conclusions: it has been confirmed that hyperspectral imaging technology possesses the capability for the non-destructive and rapid detection of soybean storage quality.

## 1. Introduction

Soybean is a significant cash crop in China, ranking among the four primary food crops alongside corn, wheat, and rice [1]. Due to the seasonality of soybean production and the continuity of consumption, storage plays a crucial role in soybean processing and supply chain [2]. However, the rich nutrients and special grain structure of soybeans render them susceptible to moisture absorption, protein denaturation, and oil rancidity during storage, leading to degradation in quality and utility [3,4]. Consequently, maintaining the quality and stability of soybeans is imperative. In China, in the national standard of GB/T 31785-2015 (soybean storage quality judgment rules) [5], the evaluation is mainly based on the determination of color, odor, crude fatty acid value, and protein solubility ratio indicators. The crude fatty acid value serves as a critical indicator of storage quality, reflecting lipid oxidation and degradation processes that are intrinsically linked to other essential parameters for assessing storage quality. Elevated crude fatty acid values are often associated with increased moisture content, which promotes enzymatic activity and microbial proliferation, thereby accelerating soybean quality deterioration. Furthermore, lipid oxidation products can interact with proteins, resulting in protein denaturation and diminished functional properties. These interrelationships underscore the reliability of crude fatty acid value as a comprehensive proxy for evaluating overall storage quality in soybeans [6,7,8].

Hyperspectral imaging technology (HSI) represents an innovative detection modality in the agricultural sector [9]. In the past several years, it has achieved promising outcomes in the realms of crop variety identification, quality assessment, and grading classification of agricultural produce [10,11]. HSI is a technology that integrates digital imaging with spectral analysis, enabling the simultaneous acquisition of image and spectral data from the sample under examination. This technology facilitates the visualization of the sample’s constituent components and its physicochemical properties [12,13,14,15]. The application of HSI technology for the evaluation of grain quality and safety has been reviewed, encompassing aspects such as nutrient content, fungal contamination, variety identification, and seed quality [16]. HSI combined with Artificial Intelligence (AI) technology has been effectively utilized for the non-destructive detection of internal defects, ripeness, and spoilage in fruits, as well as for the identification of contaminants and adulterants in meat and seafood products [17]. Hyperspectral remote sensing also plays an important role in environmental monitoring and exploration, providing important data for environmental protection by assessing vegetation health, mapping wetlands, and monitoring ecosystems [18]. In the field of precision agriculture, HSI serves as a powerful tool for real-time applications to address critical agricultural challenges such as disease detection, crop monitoring, soil mineralogy analysis, yield estimation, and classification tasks. By enabling non-destructive and high-resolution data acquisition, HSI significantly reduces the reliance on human labor and material resources for farmland management [19].

While our previous research has explored the use of HSI technology for analyzing corn fatty acid value and advanced its application in non-destructive testing of corn quality [15], this article refocuses our efforts on soybeans, another key agricultural product. Specifically, we will investigate the trends in the crude fatty acid values of soybeans under accelerated aging conditions. Compared with previous work, this article extends the research by broadening the scope from corn to soybeans, and systematically integrates spectral preprocessing, feature selection, and predictive modeling techniques to assess the storage quality of soybeans using chemical indicators like crude fatty acid values. This particular application scenario has not yet been explored in HSI-based analysis. Furthermore, our study has confirmed a strong positive correlation between storage duration and crude fatty acid values.

Although preprocessing techniques such as 1ST, 2ND, MSC, and SNV, along with feature selection methods like VISSA, CARS, and SPA, have been individually studied previously, their integration into the 1ST-VISSA-SVM model offers a highly effective approach for inspecting soybean quality. To the best of our knowledge, this particular combination of techniques has not been utilized in hyperspectral analysis of soybean storage quality until now. This model has exhibited outstanding accuracy in quantifying crude fatty acid values, achieving a coefficient of determination (R²) of 0.9888 for the test set and a root mean square error (RMSE) of 0.1857, thus outshining other models in performance. In addition, the model can visualize the spatial distribution of crude fatty acid values in soybean samples, reinforcing the practicality of HSI technology for real-time quality monitoring during storage. It offers a fast and non-destructive method for assessing soybean quality, thereby further advancing the application and development of HSI technology in the agricultural sector.

## 2. Results and Discussion

### 2.1. Changes in Crude Fatty Acid Values of Soybeans During Aging and Sample Set Partitioning

**Figure 1:**
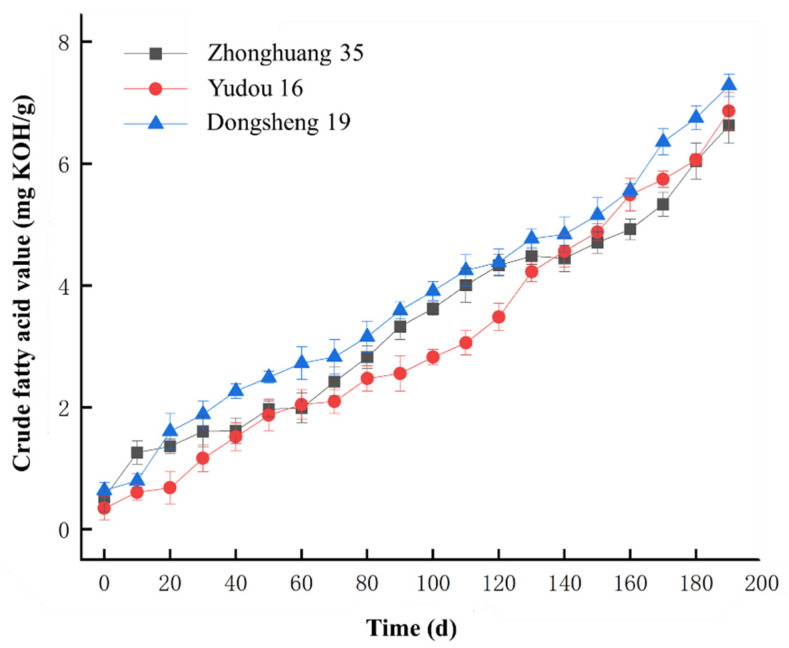
Change in crude fatty acid values during soybean aging.

Figure 1 illustrates the trend of crude fatty acid values during soybean aging. Given the substantial sample size, each data point in the figure represents the mean value of the crude fatty acid values across the collected soybean samples.

As shown in Figure 1, the crude fatty acid values for the three soybean varieties exhibited an upward trend with increasing aging time, in alignment with the previously reported trends for crude fat values [20,21,22]. At the onset of aging, the initial crude fatty acid values of the three soybean varieties were low. Specifically, Zhonghuang 35 had a value of 0.52 mg KOH/g, Yudou 16 had 0.34 mg KOH/g, and Dongsheng 19 had 0.64 mg KOH/g. After 190 days of aging, these values increased significantly to 6.6 mg KOH/g for Zhonghuang 35, 6.9 mg KOH/g for Yudou 16, and 7.3 mg KOH/g for Dongsheng 19, representing increases of 6.08 mg KOH/g, 6.56 mg KOH/g, and 6.66 mg KOH/g, respectively. Throughout the aging process of the soybean samples, the high-temperature and high-humidity environment facilitated the oxidative degradation of lipids such as triglycerides by lipases in soybeans. This resulted in an increase in crude fatty acid values, demonstrating a strong positive correlation between these values and storage duration in soybeans [23,24,25]. Therefore, the crude fatty acid values served as a reliable indicator of the soybean aging time.

The Sample Partitioning Based on Joint X-Y Distance (SPXY) algorithm is a method for dividing datasets into training and testing sets by considering both the feature space (X) and the response variable (Y), effectively leveraging both input and output information to achieve more representative data splits [26,27]. Based on the SPXY algorithm, the samples were partitioned into training and test sets at a ratio of 3:1, resulting in 225 samples allocated to the training set and 75 samples to the test set. The crude fatty acid values for soybean samples in the training set ranged from 0.33 to 7.30 mg KOH/g, while those in the test set varied between 0.34 and 7.29 mg KOH/g. Notably, the data range for the test set fell entirely within the scope of the training set, suggesting a logical and appropriate division [15,28]. The detailed results are presented in Table 1.

### 2.2. Data Extraction and Preprocessing

**Figure 2:**
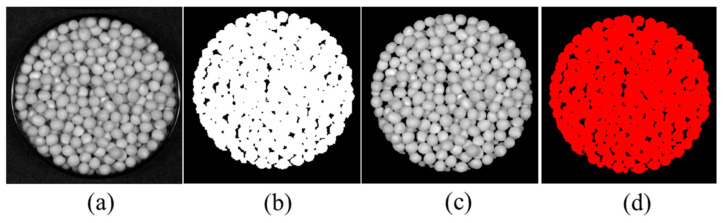
Extraction process of spectral data. (**a**) Single band hyperspectral image of soybean samples; (**b**) mask processing; (**c**) masked image; (**d**) area of interest.

The ENVI (5.3) software was used to perform masking and region of interest (ROI) selection on hyperspectral images, extracting sample hyperspectral information as illustrated in Figure 2. The mean spectral data values were calculated and recorded as raw spectral (RAW) data for subsequent analysis. Figure 2a displays the single-band hyperspectral image of a Zhonghuang 35 soybean sample at the 81st wavelength, while Figure 2b shows the mask image generated using ENVI (5.3). Figure 2c presents the image after mask application, and Figure 2d depicts the ROI selection process.

**Figure 3:**
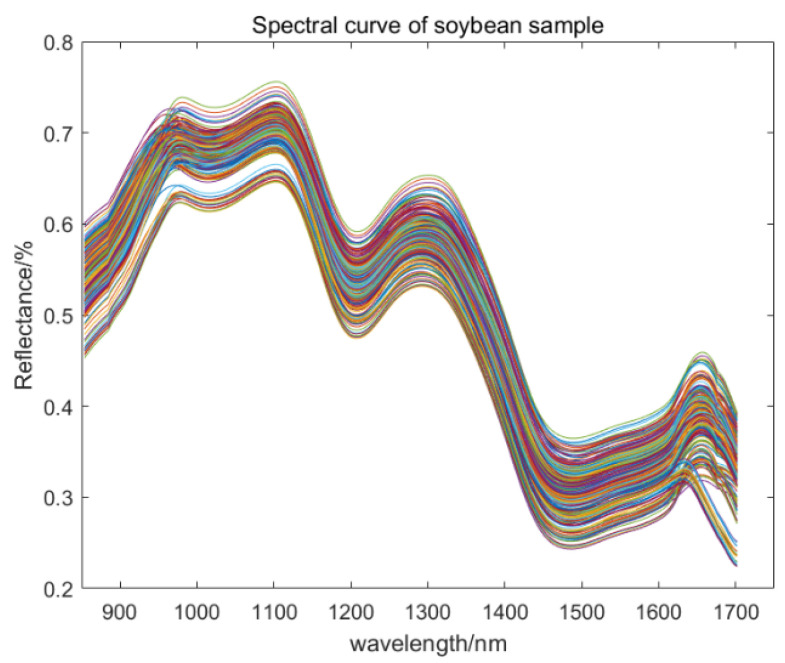
Original spectral curve of soybean samples.

Figure 3 illustrates that a total of 300 soybean samples were extracted from the original spectral dataset. During storage, soybeans undergo processes including protein denaturation and oil rancidity, which modify their moisture content and physical structure [1,21]. These changes introduce noise and interference, manifesting as variations in spectral characteristic peaks and absorption intensity.

**Figure 4:**
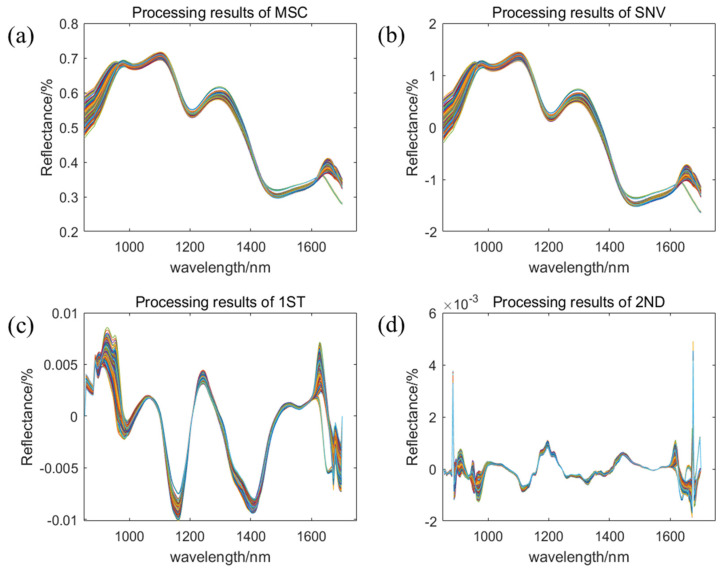
Preprocessing results of spectral information. (**a**) MSC—preprocessed spectral images; (**b**) SNV—preprocessed spectral images; (**c**) 1ST—preprocessed spectral images; (**d**) 2ND—preprocessed spectral images.

To improve the accuracy and stability of the predictive model, it is essential to eliminate redundant information through various preprocessing techniques before model development. In this study, the RAW data were subjected to preprocessing and analysis using multiplicative scatter correction (MSC), standard normal variate (SNV), first-order derivative (1ST), and second-order derivative (2ND) methods. The outcomes of these preprocessing steps are presented in Figure 4. Figure 4a displays the results of MSC preprocessing, while Figure 4b shows the outcomes of SNV preprocessing. Compared to the original spectra, both treated spectral data exhibit greater compactness, effectively reducing errors caused by variations in sample spectral scattering during acquisition. The enhanced comparability of the data is conducive to improving the accuracy of subsequent model development [29,30,31]. Figure 4c,d present the original spectra following 1ST and 2ND preprocessing, respectively. The processed spectra display more distinct trends in the curves and characteristic bands, effectively reducing background interference and enhancing the sensitivity of spectral analysis [32,33].

### 2.3. Predictive Modeling of Crude Fatty Acid Values Based on Full Band

The RAW data and spectra processed using various techniques (MSC, SNV, 1ST, 2ND) were used as independent variables, while the corresponding crude fatty acid values served as dependent variables for developing prediction models based on partial least squares regression (PLSR), support vector machine (SVM), and extreme learning machine (ELM). Table 2 presents the coefficient of determination (R^2^), root mean square error (RMSE), mean absolute error (MAE), and mean absolute percentage error (MAPE) for the models in both the training and test sets.

As shown in Table 2, the predictive performance of the PLSR, SVM, and ELM models varied following the preprocessing of spectral data. The PLSR model demonstrated the most significant enhancement in predictive performance after 1ST and 2ND preprocessing, achieving R^2^ values of 0.9748 and 0.9716 respectively, and RMSE values of 0.2778 and 0.2953 for the test set. The SVM model exhibited the greatest improvement after MSC, 1ST, and 2ND preprocessing, with all models achieving R^2^ values over 0.98 and the lowest RMSE values. Meanwhile, the ELM model performed best after 1ST preprocessing, reaching R^2^ and RMSE values of 0.9558 and 0.3680, respectively, for the test set. In conclusion, 1ST derivative preprocessing of the RAW data consistently enhanced the predictive capabilities of all three models. Consequently, 1ST preprocessing was chosen for subsequent feature band extraction and modeling analysis.

**Figure 5:**
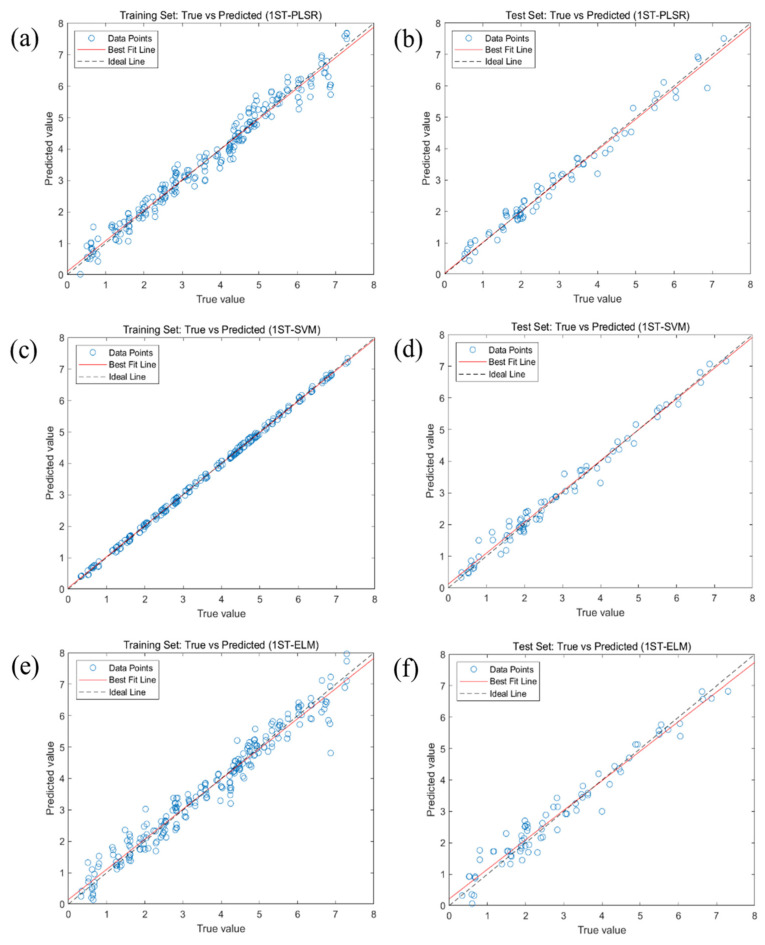
Full-band modeling results for the three models. (**a**,**b**) Full-band prediction results based on the 1ST-PLSR model; (**c**,**d**) full-band prediction results based on the 1ST-SVM model; (**e**,**f**) full-band prediction results based on the 1ST-ELM model.

Figure 5 depicts the modeling results of the PLSR, SVM, and ELM models following 1ST preprocessing of the spectral data. Specifically, Figure 5a,b display the regression performance of the 1ST-PLSR model on the training and test sets, respectively. Figure 5c,d present the prediction outcomes of the 1ST-SVM model. Similarly, Figure 5e,f respectively display the fitting efficacy of the true versus predicted values for the 1ST-ELM model. For all three models, the difference between the actual and predicted values is minimal and follows a linear distribution, indicating a relatively robust prediction performance.

### 2.4. Predictive Modeling of Crude Fatty Acid Values Based on Feature Variables

When conducting model analysis using full-band spectra, the presence of informational redundancy and an excessively high number of variables can pose a threat to model accuracy. To address these issues, this study employs three feature variable extraction techniques: variable iterative space shrinkage approach (VISSA), the successive projections algorithm (SPA), and competitive adaptive reweighted sampling (CARS). These methods effectively reduce the spectral feature dimensionality, simplifying the model structure and enhancing computational efficiency [34,35,36].

**Figure 6:**
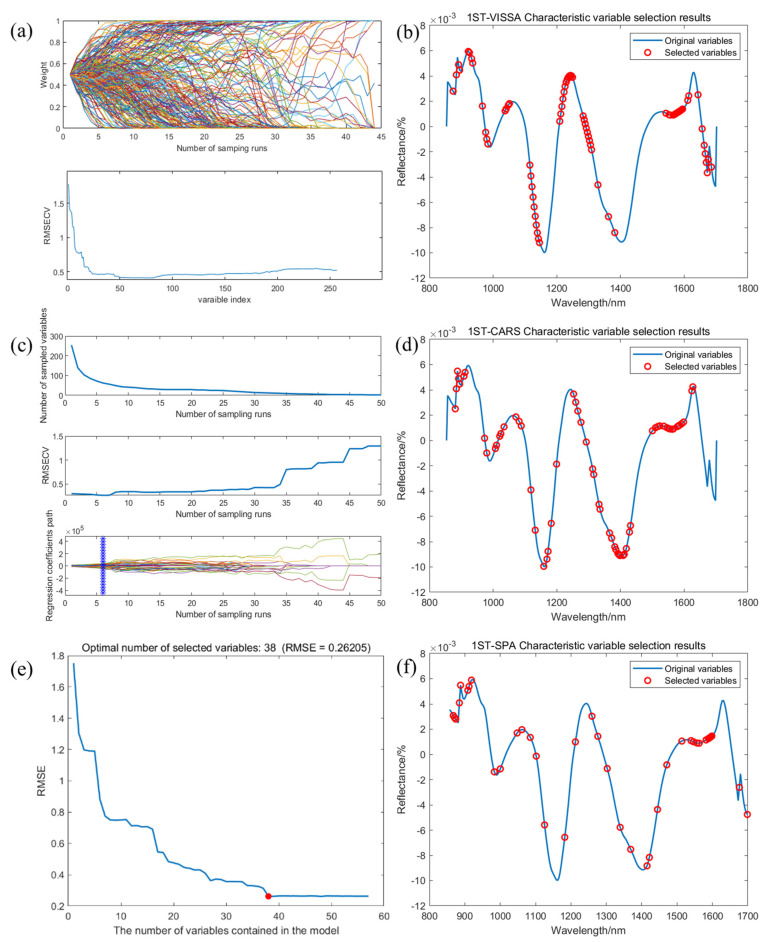
The results of extracting feature variables using three extraction methods combined with 1ST processing. (**a**) 1ST−VISSA extracts feature variable results; (**b**) distribution of characteristic variables in 1ST−VISSA; (**c**) 1ST−CARS extracts feature variable results; (**d**) distribution of characteristic variables in 1ST−CARS; (**e**) 1ST−SPA extracts feature variable results; (**f**) distribution of characteristic variables in 1ST−SPA.

Figure 6 presents the feature variable extraction results obtained using the VISSA, SPA, and CARS methods combined with 1ST processing. Figure 6a,b depict the 74 most significant variables identified by the VISSA approach, accounting for 28.91% of the total variables. Figure 6c,d present the optimal subset of 62 feature variables, as determined by the CARS algorithm after six iterations, which constitutes 24.42% of the full band. Figure 6e,f present the 38 feature variables extracted by the SPA algorithm following the elimination of redundant and interfering information, representing 14.84% of the original variables.

Feature variables extracted by the VISSA, SPA, and CARS algorithms were used as independent variables, while the corresponding crude fatty acid values served as dependent variables for developing PLSR, SVM, and ELM prediction models. The results of three models are presented in Table 3.

Table 3 demonstrates that the integration of the VISSA, SPA, and CARS feature extraction algorithms with 1ST preprocessing significantly enhances model prediction performance. Among the three algorithms, CARS had the least impact on PLSR model performance, with a test set R^2^ of 0.9729 and RMSE of 0.2883. The SPA algorithm followed, with the VISSA algorithm having the most substantial impact, although all reductions in R^2^ were within a narrow range of 0.0105. The SVM models built with feature variables from all three extraction methods showed enhanced performance, with R^2^ values surpassing 0.98. The 1ST-VISSA-SVM model emerged as the optimal one, achieving a test set R^2^ of 0.9888 and RMSE of 0.1857. The ELM model achieved its most significant performance improvement through the SPA algorithm, with the test set R^2^ increasing from 0.9558 to 0.9830.

**Figure 7:**
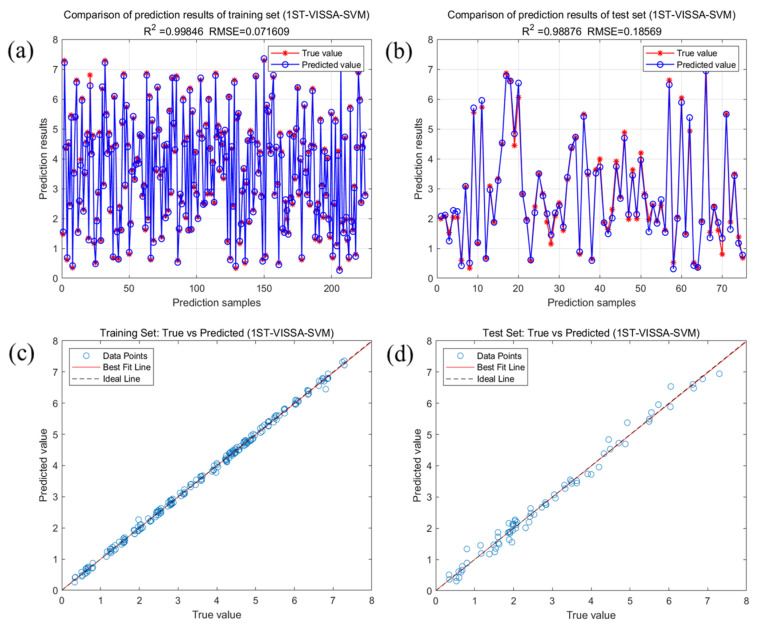
Results of the analysis of crude fatty acid values of soybeans based on the best 1ST-VISSA-SVM model. (**a**) Comparison of prediction results for the 1ST-VISSA-SVM training set; (**b**) comparison of prediction results for the 1ST-VISSA-SVM test set; (**c**) linear relationship between the predicted and true values of the training set; (**d**) linear relationship between the predicted and true values of the test set.

In conclusion, the SVM model that uses 74 feature variables extracted by the VISSA algorithm after 1ST processing of spectral data (1ST-VISSA-SVM) exhibits superior performance. Figure 7 presents a comparison of the predicted values against the actual values, along with the fitting results for the training and test sets of the optimal model. Figure 7a presents the training set results of the 1ST-VISSA-SVM model for crude fatty acid values, with a training set R^2^ of 0.9985 and RMSE of 0.0716. Figure 7b shows the test set results of the 1ST-VISSA-SVM model, with a test set R^2^ of 0.9888 and RMSE of 0.1857. As observed in Figure 7c,d, the 1ST-VISSA-SVM model exhibits high predictive capability for crude fatty acid values. The true and predicted values in both the training and test sets show minimal difference, with the data points primarily aligned along the y = x true curve.

### 2.5. Visualization of Crude Fatty Acid Values

**Figure 8:**
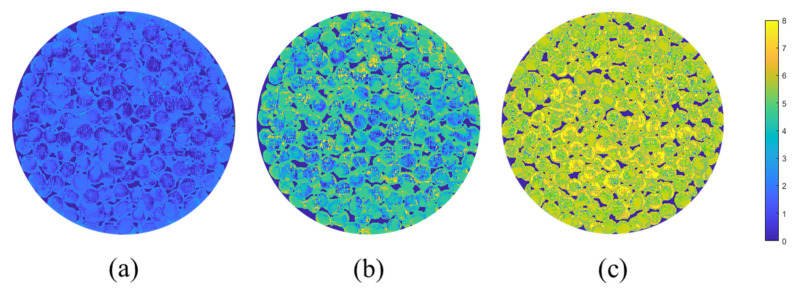
Visual distribution of crude fatty acid values of soybeans. (**a**) Visual distribution of Dongsheng 19 soybean sampled for the third time; (**b**) visual distribution of Yudou 16 soybean sampled for the 11th time; (**c**) visual distribution of Zhonghuang 35 soybean sampled for the 18th time.

The 1ST-VISSA-SVM model was identified as the optimal model for predicting crude fatty acid values of soybeans following analysis and comparison. Spectral information was extracted from each pixel in hyperspectral images of three gradient soybean samples using the ENVI (5.3) software, enabling accurate prediction of corresponding crude fatty acid values by the optima model. The predicted crude fatty acid values were mapped back onto the images, transforming them into pseudo-color images as shown in Figure 8. The soybean sample images displayed a color gradient transitioning from blue to yellow, which corresponded to the range of crude fatty acid values from low to high. Figure 8a presents a visual representation of the crude fatty acid values for the Dongsheng 19 soybean sample from the 3rd sampling. The image indicates that the soybean seeds have an overall blue coloration, corresponding to a crude fatty acid value of 1.6 mg KOH/g, indicating good quality and suitability for storage. Figure 8b shows the visual representation of the Yudou 16 soybean sample from the 11th sampling. The soybean seeds in this image appear greenish, with a corresponding crude fatty acid value of 4.0 mg KOH/g, suggesting mild unsuitability for storage. Figure 8c shows the results for the Zhonghuang 35 soybean sample from the 18th sampling. The image displays a yellowish color, with a crude fatty acid value of 6.3 mg KOH/g, which signifies severe quality deterioration making the soybeans unsuitable for storage. The results indicate that varying crude fatty acid values of soybeans correspond to distinct colorations, enabling a visual estimation of crude fatty acid values directly from visual images [37,38]. Therefore, based on the visualized distribution maps of crude fatty acid values in soybeans, the quality of soybean samples within both the training and the testing datasets can be assessed swiftly and non-destructively. Additionally, future work should include validation on additional, independent samples to further evaluate the model’s robustness and generalizability.

## 3. Materials and Methods

### 3.1. Sample Processing

#### 3.1.1. Sample Preparation

The experiment involved three varieties of newly harvested soybeans in 2022: Zhonghuang 35, Yudou 16, and Dongsheng 19. After cleaning and removing impurities, the initial moisture content of the soybeans was determined to be 9.24% (*X*_0_). To achieve the desired moisture content for corresponding soybean quality (*m*), the amount of water (*W*) needed was calculated using formula 1. The calculated volume of water was then added to the soybeans in a sealed bag, which was thoroughly mixed to ensure uniform distribution. The bag was subsequently stored flat in a refrigerator at 4 °C to facilitate moisture absorption. The target moisture content was set at 12% (*X*_1_), and after the adjustment process, the measured moisture content of the soybeans was 12.07%.

(1) $$W=\frac{m\;(X_{1}-X_{0})}{100-X_{1}}$$

#### 3.1.2. Packaging and Storage

The moisture-conditioned soybeans were packed into nylon mesh bags, with each bag containing 100 g of soybeans. This resulted in 100 bags for each of the three varieties, making a total of 300 bags. For the storage experiment, the soybean samples were kept in a controlled environment incubator set at 40 °C and 90% relative humidity [39,40].

#### 3.1.3. Soybean Sampling

Samples were withdrawn at 10-day intervals throughout the storage period, with five samples per variety for a storage time of 190 days. After allowing the sample temperature to equilibrate to room temperature, hyperspectral data were collected from the soybean samples for subsequent analysis.

### 3.2. Determination of Crude Fatty Acid Values

The crude fatty acid values of soybeans were extracted using the GB/T 14488.1-2008 method [41]. Following extraction, the acid values of the extracted crude fat were quantified according to the GB 5009.229-2016 standard [42]. The measured acid values corresponded to the crude fatty acid values of soybeans. Each sample was taken five times and subjected to two parallel determinations of crude fatty acid values within each replicate to ensure data reliability.

### 3.3. Acquisition and Correction of Hyperspectral Image Information

**Figure 9:**
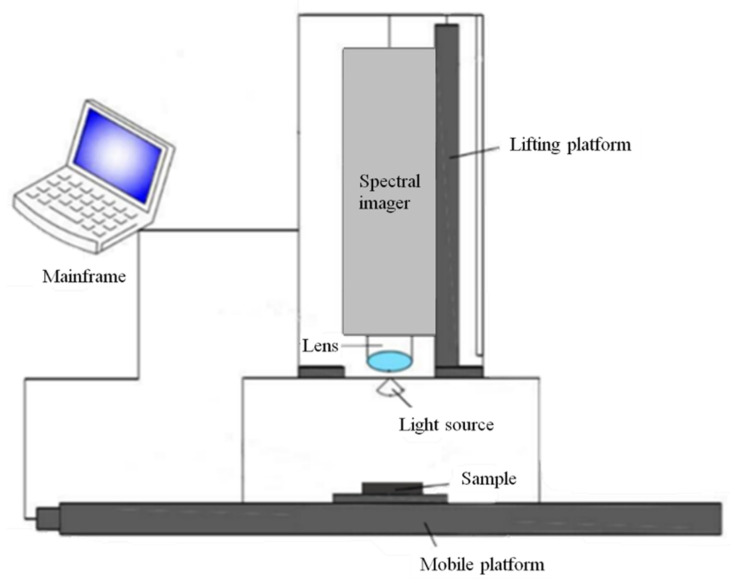
Schematic diagram of hyperspectral imaging system.

The Gaia Sorter-Dual near-infrared hyperspectral imaging system (Sichuan Shuangli Hepu Technology Co., Ltd., Chengdu, China) was used to collect the information, which mainly consists of a mainframe, a spectral imager (ImspectrorN17E Spectra Imaging Co., Ltd., Oulu, Finland), a lifting platform, a lens, a halogen light source, and an electric mobile platform, as shown in Figure 9. The system is capable of spectral acquisition within the range of 853.7–1701.8 nm, with a spectral resolution of 5 nm, comprising a total of 256 bands.

Prior to data acquisition, the hyperspectral imaging system was activated and allowed to warm up for 30 min. Soybean samples were then evenly distributed in a 90 mm diameter Petri dish. The Spec View software (Version 3.2.1, Spectral Imaging Ltd., San Jose, CA, USA) was used to operate the hyperspectral imager, and, after aligning the lens with the soybean sample, the camera height was adjusted and the lens rotated to achieve optimal focus for image capture [29,43]. The acquisition parameters were set as follows: camera height of 135 mm, exposure time of 0.042 s, electronically controlled moving platform of 50 mm/s, and frame rate of 5 frames per second.

During hyperspectral image acquisition, variations in light source intensity across different bands and the potential presence of dark current in the camera can result in noisy images. To mitigate the effects of light source fluctuations and environmental noise, the raw image (*R*_0_) must undergo black and white plate correction [10,12]. The blackboard image (*R_B_*) is acquired by masking the lens, while the whiteboard image (*R_W_*) is captured by directing the lens towards a PTFE plate with a reflectivity of nearly 100%. The corrected image *R* is then computed using the following formula.

(2) $$R=\frac{R_{0}-R_{B}}{R_{W}-R_{B}}\times 100\%$$

### 3.4. Data Analysis

Spectral data were extracted from ENVI (Version 5.3, Exelis Visual Information Solutions, Boulder, CO, USA) using image resizing, masking, and region of interest (ROI) selection methods [16]. Subsequently, the average spectral information was documented and preserved to obtain the raw spectral (RAW) dataset. In this study, the RAW data of 300 soybean samples were preprocessed using four methods: standard normal variate (SNV), first-order derivative (1ST), second-order derivative (2ND), and multiplicative scatter correction (MSC). SNV is a scatter correction technique that standardizes each spectrum by subtracting its mean and dividing by its standard deviation. This process helps reduce the impact of light scattering and path-length variations, making spectral data more comparable and analytically accurate [44]. 1ST and 2ND pertain to mathematical operations that analyze the rate of change of spectral signals. 1ST derivative is effective in eliminating baseline drift, which may be attributed to factors such as equipment, environmental conditions, or sample characteristics. It enhances spectral resolution by emphasizing the rate of change of spectral intensity and facilitating the separation of closely spaced spectral peaks [45]. In addition, 2ND builds upon the 1ST derivative to further refine peak separation and improve resolution. This enhanced resolution makes the 2ND derivative more sensitive to chemical information, allowing for more accurate analysis and identification. MSC enhances the consistency of spectral data across different samples by aligning individual spectra closer to the mean spectrum [46]. These methods were applied to eliminate noise and interference from the sample spectral information, thereby enhancing the distinctiveness of the spectral curve characteristics.

Feature variables were extracted by utilizing the variable iterative space shrinkage approach (VISSA), successive projections algorithm (SPA), and competitive adaptive reweighted sampling (CARS) to remove the interference of extraneous variables. VISSA utilizes an adaptive wavelength selection mechanism that dynamically updates variable weights, effectively preserving critical wavelengths while eliminating irrelevant ones [47]. SPA is a forward selection method that minimizes collinearity among variables by iteratively selecting wavelengths with the least redundancy. CARS identifies key wavelengths through an iterative process that retains variables with significant regression coefficients in the partial least squares (PLS) model, thereby effectively pinpointing the most relevant spectral features [48].

Partial least squares regression (PLSR), support vector machine (SVM), and extreme learning machine (ELM) models were developed in MATLAB (Version R2020a, MathWorks, Inc., Natick, MA, USA) to predict crude fatty acid values for the non-destructive assessment of soybean storage quality [44]. PLSR is a multivariate regression technique that projects both predictor and response variables into a new latent space, maximizing the covariance between them. It is particularly useful for handling multicollinearity in spectral data [49]. SVM is a supervised learning model that constructs hyperplanes in a high-dimensional space for classification or regression tasks. ELM is a single-hidden-layer feedforward neural network that delivers fast learning speeds and strong generalization capabilities [50].

### 3.5. Visualization of Crude Fatty Acid Values

Pixel points were extracted from the hyperspectral image data for the third sample of Dongsheng 19, the 11th sample of Yudou 16, and the 18th sample of Zhonghuang 35 soybean varieties. These points were then used to predict crude fatty acid values using the optimized model, generated in a novel data matrix. Subsequently, pseudo-color maps were generated to showcase the dynamic distribution of the indicator. The maps use color gradients to represent the magnitude of the values, where higher values appear in shades of yellow and lower values in shades of blue.

## 4. Conclusions

Hyperspectral imaging technology, in conjunction with PLSR, SVM, and ELM machine learning methods, effectively predicted the dynamics of crude fatty acid values of soybeans undergoing accelerated aging. This approach offers a reliable and efficient strategy for the detection and monitoring of soybean storage quality. The 1ST-VISSA-SVM model exhibited the superior prediction performance for the crude fatty acid values of soybeans, with test set coefficients of determination and root-mean-square errors of 0.9888 and 0.1857. Furthermore, the 1ST-VISSA-SVM model enabled the spatial visualization of the chemical distribution of crude fatty acid values, allowing intuitive assessment of their relative levels in soybean samples across the dataset. We believe that HSI technology holds significant capacity not only for evaluating soybean grain quality but also for broader applications in the production and processing of other food products.

## Figures and Tables

**Table 1:** Sample Partitioning Results for SPXY.

Data Set	Sample Size	Crude Fatty Acid Value (mg KOH/g)			
		Maximum	Minimum	Average Value	Standard Deviation
Training set	225	7.30	0.33	3.62	1.83
Test set	75	7.29	0.34	2.81	1.76

**Table 2:** Modeling results based on the full band.

Model	Pretreatment	Training Set				Test Set			
		R^2^	RMSE	MAE	MAPE	R^2^	RMSE	MAE	MAPE
PLSR	RAW	0.9615	0.3579	0.2769	0.1270	0.9626	0.3385	0.2581	0.1704
	MSC	0.9623	0.2731	0.3541	0.1219	0.9522	0.3828	0.2933	0.2076
	SNV	0.9611	0.2823	0.3600	0.1303	0.9505	0.3899	0.2996	0.2130
	1ST	0.9712	0.2403	0.3095	0.1073	0.9748	0.2778	0.2205	0.1467
	2ND	0.9882	0.1535	0.1986	0.0759	0.9716	0.2953	0.2525	0.1553
SVM	RAW	0.9905	0.1780	0.0978	0.0402	0.9442	0.4138	0.3310	0.2094
	MSC	0.9987	0.0644	0.0662	0.0288	0.9838	0.2230	0.1612	0.0987
	SNV	0.9987	0.0648	0.0664	0.0287	0.9738	0.2838	0.1936	0.1020
	1ST	0.9988	0.0614	0.0642	0.0283	0.9826	0.2310	0.1714	0.0973
	2ND	0.9987	0.0630	0.0653	0.0286	0.9802	0.2463	0.1782	0.1105
ELM	RAW	0.9134	0.5368	0.4121	0.2086	0.8365	0.7084	0.4874	0.3889
	MSC	0.9376	0.3741	0.4555	0.1895	0.9251	0.4792	0.3904	0.2911
	SNV	0.9661	0.2554	0.3359	0.1195	0.9438	0.4154	0.3205	0.2195
	1ST	0.9599	0.2750	0.3651	0.1241	0.9558	0.3680	0.2921	0.1947
	2ND	0.9216	0.3963	0.5107	0.1954	0.9062	0.5364	0.4035	0.2610

**Table 3:** Modeling results of feature variables under 1ST processing.

Model	Feature Extraction	Training Set				Test Set			
		R^2^	RMSE	MAE	MAPE	R^2^	RMSE	MAE	MAPE
PLSR	None	0.9712	0.3095	0.1073	0.2403	0.9748	0.2778	0.1467	0.2205
	VISSA	0.9648	0.3422	0.2617	0.1229	0.9643	0.3311	0.2530	0.1673
	SPA	0.9739	0.2947	0.2323	0.1048	0.9699	0.3040	0.2424	0.1602
	CARS	0.9772	0.2757	0.2173	0.1078	0.9729	0.2883	0.2311	0.1620
SVM	None	0.9988	0.0642	0.0283	0.0614	0.9826	0.2310	0.0973	0.1714
	VISSA	0.9985	0.0716	0.0631	0.0281	0.9888	0.1857	0.1409	0.0805
	SPA	0.9970	0.0996	0.0759	0.0342	0.9881	0.1908	0.1423	0.0864
	CARS	0.9980	0.0806	0.0681	0.0290	0.9847	0.2167	0.1686	0.0928
ELM	None	0.9599	0.3651	0.1241	0.2750	0.9558	0.3680	0.1947	0.2921
	VISSA	0.9899	0.1836	0.1466	0.0664	0.9790	0.2537	0.1953	0.1075
	SPA	0.9928	0.1545	0.1200	0.0564	0.9830	0.2286	0.1744	0.1166
	CARS	0.9898	0.1839	0.1392	0.0558	0.9770	0.2655	0.2046	0.1158

## Data Availability

The data presented in this study are available on request from the corresponding author.

## References

[1] Wijewardana C., Reddy K.R., Bellaloui N. (2019). Soybean seed physiology, quality, and chemical composition under soil moisture stress. Food Chem..

[2] Zhu Z., Chen S., Wu X., Xing C., Yuan J. (2018). Determination of soybean routine quality parameters using near-infrared spectroscopy. Food Sci. Nutr..

[3] Vergara R., Silva R.N.O.D., Nadal A.P., Gadotti G.I., Aumonde T.Z., Villela F.A. (2019). Harvest delay, storage and physiological quality of soybean seeds. J. Seed Sci..

[4] Weerasekara I., Sinniah U.R., Namasivayam P., Nazli M.H., Abdurahman S.A., Ghazali M.N. (2021). The influence of seed production environment on seed development and quality of soybean (*Glycine max* (L.) Merrill). Agronomy.

[5] (2015). Guidelines for Evaluation of Soybean Storage Character.

[6] Song W., Sun S., Wu T., Yang R., Tian S., Xu C., Jiang B., Yuan S., Hou W., Wu C. (2023). Geographic distributions and the regionalization of soybean seed compositions across China. Food Res. Int..

[7] Fu D., Zhou J., Scaboo A.M., Niu X. (2021). Nondestructive phenotyping fatty acid trait of single soybean seeds using reflective hyperspectral imagery. J. Food Process Eng..

[8] Oner F., Aykutlu H.M. (2019). The effect of maize-soybean intercropping systems on a set of technological and physiological properties. Appl. Ecol. Environ. Res..

[9] Aviara N.A., Liberty J.T., Olatunbosun O.S., Shoyombo H.A., Oyeniyi S.K. (2022). Potential application of hyperspectral imaging in food grain quality inspection, evaluation and control during bulk storage. J. Agric. Food Res..

[10] An D., Zhang L., Liu Z., Liu J., Wei Y. (2023). Advances in infrared spectroscopy and hyperspectral imaging combined with artificial intelligence for the detection of cereals quality. Crit. Rev. Food. Sci. Nutr..

[11] Shi T., Gao Y., Song J., Ao M., Hu X., Yang W., Chen W., Liu Y., Feng H. (2024). Using VIS-NIR hyperspectral imaging and deep learning for non-destructive high-throughput quantification and visualization of nutrients in wheat grains. Food Chem..

[12] Wu J., Zhang Y., Hu P., Wu Y. (2024). A review of the application of hyperspectral imaging technology in agricultural crop economics. Coatings.

[13] Wang B., Sun J., Xia L., Liu J., Wang Z., Li P., Guo Y., Sun X. (2023). The applications of hyperspectral imaging technology for agricultural products quality analysis: A review. Food Res. Int..

[14] Teet S.E., Hashim N. (2023). Recent advances of application of optical imaging techniques for disease detection in fruits and vegetables: A review. Food Control.

[15] Zhang Y., Liu S., Zhou X., Cheng J. (2024). Study on rapid non-destructive detection method of corn freshness based on hyperspectral imaging technology. Molecules.

[16] Lu B., Dao P.D., Liu J., He Y., Shang J. (2020). Recent advances of hyperspectral imaging technology and applications in agriculture. Remote Sens..

[17] Nikzadfar M., Rashvand M., Zhang H., Shenfield A., Genovese F., Altieri G., Matera A., Tornese I., Laveglia S., Paterna G. (2024). Hyperspectral imaging aiding artificial intelligence: A reliable approach for food qualification and safety. Appl. Sci..

[18] Pour A.B., Zoheir B., Pradhan B., Hashim M. (2021). Editorial for the special issue: Multispectral and hyperspectral remote sensing data for mineral exploration and environmental monitoring of mined areas. Remote Sens..

[19] Ram B.G., Oduor P., Igathinathane C., Howatt K., Sun X. (2024). A systematic review of hyperspectral imaging in precision agriculture: Analysis of its current state and future prospects. Comput. Electron. Agric..

[20] Desta K.T., Hur O.S., Lee S., Yoon H., Shin M.J., Yi J., Lee Y., Ro N.Y., Wang X., Choi Y.M. (2022). Origin and seed coat color differently affect the concentrations of metabolites and antioxidant activities in soybean (*Glycine max* (L.) Merrill) seeds. Food Chem..

[21] Abdelghany A.M., Zhang S., Azam M., Shaibu A.S., Feng Y., Li Y., Tian Y., Hong H., Li B., Sun J. (2020). Profiling of seed fatty acid composition in 1025 Chinese soybean accessions from diverse ecoregions. Crop. J..

[22] Li Y., Yu Z., Jin J., Zhang Q., Wang G., Liu C., Wu J., Wang C., Liu X. (2018). Impact of elevated CO_2_ on seed quality of soybean at the fresh edible and mature stages. Front. Plant Sci..

[23] Ebone L.A., Caverzan A., Tagliari A., Chiomento J.L.T., Silveira D.C., Chavarria G. (2020). Soybean seed vigor: Uniformity and growth as key factors to improve yield. Agronomy.

[24] Souza A., Santos D., Rodrigues A.A., Zuchi J., Vieira M.C., Sales J.F. (2023). Physical and physiological soybean seed qualities stored under different environmental conditions and storage bag depths. Braz. J. Biol..

[25] Prabakaran M., Lee K., An Y., Kwon C., Kim S., Yang Y., Ahmad A., Kim S., Chung I. (2018). Changes in soybean (*Glycine max* L.) flour fatty-acid content based on storage temperature and duration. Molecules.

[26] Luo W., Zhang J., Liu S., Huang H., Zhan B., Fan G., Zhang H. (2024). Prediction of soluble solid content in Nanfeng mandarin by combining hyperspectral imaging and effective wavelength selection. J. Food Compos. Anal..

[27] Shao Y., Liu Y., Xuan G., Shi Y., Li Q., Hu Z. (2022). Detection and analysis of sweet potato defects based on hyperspectral imaging technology. Infrared Phys. Technol..

[28] Zhang Y., Lu G., Zhou X., Cheng J.H. (2022). Non-destructive hyperspectral imaging for rapid determination of catalase activity and ageing visualization of wheat stored for different durations. Molecules.

[29] Wang Y., Ou X., He H.J., Kamruzzaman M. (2024). Advancements, limitations and challenges in hyperspectral imaging for comprehensive assessment of wheat quality: An up-to-date review. Food Chem. X.

[30] Jiang X., Bu Y., Han L., Tian J., Hu X., Zhang X., Huang D., Luo H. (2023). Rapid nondestructive detecting of wheat varieties and mixing ratio by combining hyperspectral imaging and ensemble learning. Food Control.

[31] Dashti A., Mueller-Maatsch J., Roetgerink E., Wijtten M., Weesepoel Y., Parastar H., Yazdanpanah H. (2023). Comparison of a portable VIS-NIR hyperspectral imaging and a snapscan SWIR hyperspectral imaging for evaluation of meat authenticity. Food Chem. X.

[32] Ram B.G., Zhang Y., Costa C., Ahmed M.R., Peters T., Jhala A., Howatt K., Sun X. (2023). Palmer amaranth identification using hyperspectral imaging and machine learning technologies in soybean field. Comput. Electron. Agric..

[33] Cozzolino D., Williams P.J., Hoffman L.C. (2023). An overview of pre-processing methods available for hyperspectral imaging applications. Microchem. J..

[34] Dai Y., Yan B., Xiong F., Bai R., Wang S., Guo L., Yang J. (2024). Tanshinone content prediction and geographical origin classification of *Salvia miltiorrhiza* by combining hyperspectral imaging with chemometrics. Foods.

[35] Zhang J., Lei Y., He L., Hu X., Tian J., Chen M., Huang D., Luo H. (2023). The rapid detection of the tannin content of grains based on hyperspectral imaging technology and chemometrics. J. Food Compos. Anal..

[36] Wang Y., Zhang Y., Yuan Y., Zhao Y., Nie J., Nan T., Huang L., Yang J. (2022). Nutrient content prediction and geographical origin identification of red raspberry fruits by combining hyperspectral imaging with chemometrics. Front. Nutr..

[37] Song Y., Cao S., Chu X., Zhou Y., Xu Y., Sun T., Zhou G., Liu X. (2023). Non-destructive detection of moisture and fatty acid content in rice using hyperspectral imaging and chemometrics. J. Food Compos. Anal..

[38] Aulia R., Amanah H.Z.Z., Lee H., Kim M.S.S., Baek I., Qin J., Cho B. (2023). Protein and lipid content estimation in soybeans using Raman hyperspectral imaging. Front. Plant Sci..

[39] Zaaboul F., Zhao Q., Xu Y., Liu Y. (2022). Soybean oil bodies: A review on composition, properties, food applications, and future research aspects. Food Hydrocoll..

[40] Jo H., Noy N., Song J.T., Lee J. (2023). Selection of soybean accessions with seed storability test under accelerated aging conditions. Plant Breed. Biotechnol..

[41] (2008). Oilseeds: Determination of Oil Content.

[42] (2016). National Food Safety Standard: Determination of Acid Value in Food.

[43] Zhu J., Li H., Rao Z., Ji H. (2023). Identification of slightly sprouted wheat kernels using hyperspectral imaging technology and different deep convolutional neural networks. Food Control.

[44] Zuo J., Peng Y., Li Y., Zou W., Chen Y., Huo D., Chao K. (2023). Nondestructive detection of nutritional parameters of pork based on NIR hyperspectral imaging technique. Meat Sci..

[45] Femenias A., Gatius F., Ramos A.J., Sanchis V., Marin S. (2021). Near-infrared hyperspectral imaging for deoxynivalenol and ergosterol estimation in wheat samples. Food Chem..

[46] Tian P., Meng Q., Wu Z., Lin J., Huang X., Zhu H., Zhou X., Qiu Z., Huang Y., Li Y. (2023). Detection of mango soluble solid content using hyperspectral imaging technology. Infrared Phys. Technol..

[47] Zhang L., Sun J., Zhou X., Nirere A., Wu X., Dai R. (2020). Classification detection of saccharin jujube based on hyperspectral imaging technology. J. Food Process Preserv..

[48] Feng Z., Wang L., Yang Z., Zhang Y., Li X., Song L., He L., Duan J., Feng W. (2022). Hyperspectral monitoring of powdery mildew disease severity in wheat based on machine learning. Front. Plant Sci..

[49] Zhang L., Wang Y., Wei Y., An D. (2022). Near-infrared hyperspectral imaging technology combined with deep convolutional generative adversarial network to predict oil content of single maize kernel. Food Chem..

[50] Zhu H., Chu B., Zhang C., Liu F., Jiang L., He Y. (2017). Hyperspectral imaging for presymptomatic detection of tobacco disease with successive projections algorithm and machine-learning classifiers. Sci. Rep..

